# Relapse of acute myeloid leukemia after allogeneic stem cell transplantation: immune escape mechanisms and current implications for therapy

**DOI:** 10.1186/s12943-023-01889-6

**Published:** 2023-11-11

**Authors:** Tatjana Sauerer, Giuliano Filippini Velázquez, Christoph Schmid

**Affiliations:** Department of Hematology and Oncology, Augsburg University Hospital and Medical Faculty, Bavarian Cancer Research Center (BZKF) and Comprehensive Cancer Center Augsburg, Augsburg, Germany

**Keywords:** Acute myeloid leukemia, Immune escape, Allogeneic stem cell transplantation, Relapse, Human leukocyte antigen, Immune checkpoints, Therapeutic options

## Abstract

Acute myeloid leukemia (AML) is a heterogeneous disease characterized by the expansion of immature myeloid cells in the bone marrow (BM) and peripheral blood (PB) resulting in failure of normal hematopoiesis and life-threating cytopenia. Allogeneic hematopoietic stem cell transplantation (allo-HCT) is an established therapy with curative potential. Nevertheless, post-transplant relapse is common and associated with poor prognosis, representing the major cause of death after allo-HCT. The occurrence of relapse after initially successful allo-HCT indicates that the donor immune system is first able to control the leukemia, which at a later stage develops evasion strategies to escape from immune surveillance. In this review we first provide a comprehensive overview of current knowledge regarding immune escape in AML after allo-HCT, including dysregulated HLA, alterations in immune checkpoints and changes leading to an immunosuppressive tumor microenvironment. In the second part, we draw the line from bench to bedside and elucidate to what extend immune escape mechanisms of relapsed AML are yet exploited in treatment strategies. Finally, we give an outlook how new emerging technologies could help to improve the therapy for these patients, and elucidate potential new treatment options.

**Supplementary Information:**

The online version contains supplementary material available at 10.1186/s12943-023-01889-6.

## Background

Acute myeloid leukaemia (AML) is a heterogeneous disease characterized by clonal expansion of immature myeloid cells in the bone marrow (BM) and peripheral blood (PB), resulting in failure of normal hematopoiesis and life-threating cytopenia [1]. For the majority of AML patients, allogeneic hematopoietic stem cell transplantation (allo-HCT) is the only treatment with curative potential [2], whereby the curative effect is mainly based on the graft-versus-leukaemia (GvL) reaction provided by the transferred immune cells, mainly T- and NK-cells [3]. Hence, allo-HCT is considered as the prototype of cellular immunotherapy. Nevertheless, the occurrence of relapse after allo-HCT is common and associated with poor prognosis [4, 5] indicating that despite initial control by the allogeneic immune system, the leukemic cells develop escape strategies over time.

Regarding treatment for relapse post allo-HCT, long-term remission is mainly achieved by treatment concepts that include any type of cellular immunotherapy, such as donor lymphocyte infusion (DLI) or a second allo-HCT (allo-HCT2) [6]. Nevertheless, these treatments are still ineffective in a large proportion of patients, most likely because the various mechanisms initially leading to evasion of the immune control and relapse are still ongoing and are not addressed by the therapeutic efforts. Beyond, in contrast to lymphoid malignancies, the enormous potential of modern immunotherapeutic strategies, such as chimeric antigen receptor (CAR) T-cells, immune checkpoint blockade (ICB) and bispecific antibodies could not yet be transferred to AML, although this disease in principle is considered as highly immune-responsive [7]. In fact, the majority of AML patients relapsing after allo-HCT will eventually die from disease progression [6]. This might however change in a near future, as modern genome and molecular based technologies are providing an unprecedented understanding of the interplay between cancer cells and the tumor microenvironment (TME). Consequently, we are now gaining critical insights about the molecular pathways connected to the immune escape mechanisms of AML relapse after allo-HCT, that are yet to be exploited by novel and specific treatment strategies. It is hoped that increasing knowledge will allow us to approach a new treatment era based on individualized and therefore hopefully more efficient immunotherapy for relapsed AML [8].

In this review, we summarize different ways of immune escape in AML patients after allo-HCT, separating mechanisms leading to impaired HLA expression from those characterized by aberrant immune checkpoint expression and differential effects leading to a remodeling of the TME. Beyond, we discuss implications for present and future therapeutic approaches and give an outlook on how new innovative technologies could help to identify new targets to overcome immune escape in relapsing AML.

### Impaired HLA expression

An intact antigen-presentation machinery is critical for an effective recognition of leukemia cells by the donor immune system. HLA possess outstanding ability to elicit an immune response either by presentation of variable peptides such as minor histocompatibility antigens (miHag) and tumor-associated antigens (TAA), or as direct target. The genomic HLA molecule disparity between donor and recipient triggers T-cell allo-recognition [9]. In contrast, donor-versus-host NK-cell alloreactivity relies on a mismatch between inhibitory receptors for self-major histocompatibility complex (MHC) class I on donor NK-cells and the respective ligands on host T-cells. Consequently, the missing expression of self-MHC class I molecules mediates recognition and attack of allogeneic targets by donor NK-cells [10, 11].

Impaired HLA expression is a quite rare event in hematologic neoplasms at time of diagnosis, but frequently occurs in patients with relapse after allo-HCT [12–15]. Hence, impaired NK- or T-cell recognition or even specific anergy caused by disturbed leukemia cell recognition following either non-genomic aberrations of HLA expression or HLA loss is one of best-described immune evasion mechanisms of AML blasts.

#### Epigenetic downregulation of HLA expression

In the setting of HLA-matched allo-HCT, non-genomic loss of HLA expression seems to play a major role for post-transplant relapse. Two seminal studies including patients transplanted from matched sibling donors (MSD), MUD, and MMUD [16] and haploidentical donors [17] observed a significant downregulation of classical HLA class II genes (HLA-DP, -DQ, and –DR) and other genes involved in antigen processing and presentation by MHC class II (CD74, IFI30, IL-16, CTSS, CIITA) in 30–50% of AML relapses post allo-HCT. As a consequence, the originally primed T-cells failed in antigen-recognition of the disease, thus contributing to escape of leukemic cells from the GvL effect and relapse. Importantly, in both studies, comparative analyses with relapse after chemotherapeutic treatment alone indicated, that this relapse mechanism might again be specifically linked to the immunological effects of allo-HCT. Beyond, HLA downregulation occurred largely non-overlapping with other mechanisms of immune escape such as dysregulation of inhibitory immune checkpoints (see next section).

The molecular mechanism behind HLA dysfunction on leukemia cells has been extensively studied. Downregulation of MHC class II transactivator CIITA (MHC2TA) by hypermethylation of its promotor has been detected by gene expression analysis [16, 17]. Beyond, the epigenetic regulator polycomb repressive complex 2 (PRC2) was involved in HLA alteration and the CIITA. Transcriptionally driven loss of HLA class II expression was associated with a PRC2-mediated decrease in chromatin accessibility, highlighting a novel epigenetic pathway of immune escape. In the same study, HLA expression could be restored by pharmacological inhibition of EZH2, the catalytic subunit of PRC2, in AML relapse in vivo and in vitro [18]. This was accompanied by a consistent rescue of anti-leukemic T-cell activity. A third mechanism of HLA downregulation comprises mouse-double-minute-2 (MDM2), which is overexpressed on malignant cells and possesses high oncogenic potential [19]. MDM2 serves as a negative regulator of the transcription factor p53 [20] and reduces intracellular p53 via inhibition of its transcription and increase of its degradation [21]. Recently, Ho et al. investigated the role of MDM2 in AML after allo-HCT and reported that MDM2 inhibition was able to restore p53 activity, leading to increased MHC class II expression and higher frequencies of non-exhausted T-cells. As a consequence, immune evasion of AML cells was counteracted and their sensitivity to allogeneic T-cell mediated cytotoxicity was restored [22]. According to preclinical date, about 2/3 AML cell lines and patient-derived samples were sensitive to MDM2 inhibition [23–25].

Whereas the studies published so far mainly comprise HLA class II molecules, recent work reported HLA aberrations also including HLA class I, which were frequently detected in relapsing AML patients [26]. Hence, both types of HLA (I and II) can be affected by somatic mutations and thus damp the anti-leukemic immune responses with the consequence of tumor immune evasion.

#### Genomic loss of HLA

During the last decade, allo-HCT from haploidentical family donors (haplo-HCT) has become a frequently used transplant strategy for allo-HCT in patients lacking a HLA matched donor [27]. In this setting, incompatible HLA molecules on leukemic blasts are the immunodominant GvL targets of alloreactive T-cells [28], bearing the risk of new immune escape strategies to develop. Genomic loss of one HLA haplotype was first described in 2009 in patients relapsing after haplo-HCT for AML and myelodysplastic syndrome (MDS) (either non-T-cell depleted or using purified CD34^+^ cells with subsequent DLI) [14]. Lack of patient-specific HLA alleles was observed on the leukemic blasts in about 30% of relapses. The underlying mechanism was an irreversible loss of genomic material on the short arm of chromosome 6, encompassing the HLA region, without numerical chromosomal alterations. In other words, all incompatible class I and II HLA molecules on the leukemic cells were permanently lost, whereby this loss was compensated by duplication of the remaining compatible haplotype, resulting in homozygosity for the shared HLA haplotype (uniparental disomy: daughter cell carrying two HLA haplotypes derived from only one parent after mitotic recombination) [29, 30]. As a direct consequence, leukemic cells became undetectable by alloreactive donor T-cells, and thus escaped from the GvL effect, conferring a selective advantage that resulted in uncontrolled proliferation and clinical relapse [14, 31]. It remained unclear why relapses were not avoided by alloreactive NK-cells. Nevertheless, this failure to control leukemia after HLA loss supports the “missing self” hypothesis [11] as a mechanism for the activation of NK alloreactivity, since in cases of uniparental dysomy, the overall expression of HLA class I molecules on leukemic cells remains unaltered, and thus the retention of the “self” HLA haplotype seems to prevent NK-cell activation [31]. Further studies investigating the incidence and outcome of patients with HLA loss after haplo-HCT reported rates of HLA loss between 25–51% [32–34], with incidences being independent from the in vivo T-cell depletion strategies (anti-thymocyte globulin [ATG] or post-transplant cyclophosphamide [pt-CY]) [35, 36]. Interestingly, HLA loss was also detected in six patients that did not experience overt hematological relapse [33].

The temporal and biological origin of HLA loss has not yet been completely elucidated. Although rare alterations involving HLA at the time of diagnosis in patients with hematological cancers were reported [12, 30], the more frequent detection of this phenomenon after allo-HCT suggests a key role of a selective immune pressure mediated by allogeneic T-cells [37]. This hypothesis is further supported by the results of Crucitti et al., who have observed a correlation between the numbers of T-cells transferred with the graft and the incidence of HLA loss [38]. Another important finding was the significant delay of relapse with HLA loss which occurred at a median time of 307 days from haplo-HCT.

Apparently, HLA loss is not exclusive of haplo-HCT and AML, but was documented in AML relapses after matched unrelated (MUD) and mismatch unrelated donor (MMUD) allo-HCT [39], and also after haplo-HCT for other haematological malignancies, including Hodgkin’s lymphoma [32] and acute lymphoblastic leukemia [34]. However, the frequencies of HLA loss vary depending on donor source, with an inverse relation to the grade of donor-recipient mismatch: In a large series, HLA loss was detected in 23%, 12%, 4% and 0% after allo-HCT from haploidentical, MMUD, MUD and unrelated cord blood (UCB) stem cell sources, respectively [15].

### Aberrant immune checkpoint expression

The understanding of immune checkpoints (ICP) has revolutionized the role of immunotherapy in cancer treatment. In healthy individuals ICP on immune effector cells represent physiological control mechanisms essential for maintaining immune tolerance and preventing autoimmunity [40].

#### PD1/PD-L1

*Programmed cell death protein 1* (PD-1) and its ligand PD-L1 constitute a major inhibitory axis. Upon engagement of these two proteins, T-cells exhibit a hyporesponsive T-cell differentiation state defined by poor effector function, sustained expression of inhibitory receptors, lack of response to stimuli and a transcriptional state distinct from that of functional effector or memory T-cells [41]. The PD-1 pathway and several other inhibitory checkpoints co-regulate T-cell exhaustion, limiting the effectiveness of T-cells against infection and cancer [40]. An aberrant expression of PD-1/PD-L1 molecules has been reported in various malignancies including AML, so far representing one of the most important immune evasion strategies exploited by cancer cells [42, 43]. PD-1 is expressed on various immune cells including different T-cell subtypes, and can bind to its ligand PD-L1 on AML blasts. Significant upregulation of PD-1 expression on BM T cells was confirmed in 42% of relapsed AML patients [44] and similar results could be obtained for levels of PD-L1 on AML blasts [17, 45]. Via the PD-1/PD-L1 axis, leukemic cells have shown to induce T-cell exhaustion and recruitment of regulatory T-cells (T_regs_). The resulting T-cell suppression was associated with AML relapse after allo-HCT [17, 46–48].

#### CTLA-4

The inhibitory receptor *cytotoxic T-lymphocyte-associated protein 4* (CTLA-4) is an ICP molecule that binds to the ligands CD80 and CD86 in a competitive manner with CD28. Normally, CD80 and CD86 interact with CD28 to provide a co-stimulatory signal to T-cell receptor (TCR) mediated activation. In contrast, interactions of these ligands with CTLA-4 antagonize CD28-mediated co-stimulation and inhibit T-cell responses [49]. The biology of CTLA-4 is particular in a way that it is predominantly found in intracellular vesicles (90%) of T_reg_ cells or activated conventional T-cells due to active endocytosis from the plasma membrane, from where it can be recycled to the surface or degraded in lysosomal compartments [49]. CD80 and CD86 has been found upregulated in AML patients after relapse [17, 47], further supporting a contribution of the CD80/CD86-CTLA-4 axis to immune evasion in AML. However, results of CTLA-4 expression remain conflicting as Noviello et al. found significant increased CLTA-4 on T cells of relapsed AML patients [47] whereas another study could not confirm any CLTA-4 changes between different clinical stages [17].

#### TIM-3

Apart from PD-1/PD-L1 and CTLA-4, *T-cell immunoglobulin and mucin domain 3* (TIM-3), which physiologically binds galectin-9 (Gal-9), is highly expressed on AML blasts, and plays a major role in immune escape of AML and relapse post allo-HCT [50]. Interestingly, TIM-3 is also expressed on leukemic stem (LSCs) and progenitor cells of other myeloid malignancies, but not on normal hematopoietic stem cells [51]. Gal-9 is not only involved in impaired T-and NK-cell function but is also essential in maintaining LSCs via an autocrine signaling loop involving TIM-3 on LSCs, promoting their self-renewal [52, 53]. Kong et al. showed in immunophenotypic analyses of AML patients that high PD-1/TIM-3 expression on CD8^+^ T-cells was strongly associated with T-cell exhaustion and relapse post allo-HCT, whereby PD-1/TIM-3 positive T-cells of PB were already detected 2 months before clinical relapse, suggesting a predictive value of this observation [54]. Additionally, increased levels of BM CD8^+^ T-cells from relapsing AML patients expressing TIM-3, PD-1 and CTLA-4, have been reported, while their corresponding ligands Gal-9, PDL-1, CD80, and CD86 were expressed on leukemic blasts [55].

#### TIGIT

*T-cell immunoreceptor with Ig and ITIM domains* (TIGIT) is another inhibitory receptor present on NK and T-cells. Ligands for TIGIT are the poliovirus receptors (PVR; also known as CD155 and nectin-like protein 5) and PVRL2 (CD112; nectin-2). TIGIT binds to these ligands in competition with the activating costimulatory receptor DNAM-1, thereby suppressing the activity of NK and T-cells [56, 57]. Various studies demonstrated a significantly higher expression of TIGIT on infiltrating T-cells in the BM of relapsed AML patients after allo-HCT compared to non-relapsed patients. This expression was associated with poor clinical outcome, suggesting TIGIT as a biomarker for immune escape in AML and potential therapeutic target [48, 58, 59]. This goes in line with a recently conducted work by Gournay et al., who reported that high levels of TIGIT on CD4^+^ T-cells early after allo-HCT are associated with AML relapse [60]. TIGIT, TIM-3, and LAG-3 were also highly expressed on immunosuppressive tumor-associated macrophages (TAMs) of AML patients in active disease [61]. Moreover, both ligands of TIGIT, PVRL2 (CD112) and PVR (CD155) were also reported to be upregulated on AML blasts at relapse after allo-HCT compared to the level at diagnosis, which was significantly associated with poorer clinical outcome [17, 58, 62].

#### KLRG-1

Another checkpoint, *KLRG-1* was reported to be involved in AML relapses post allo-HCT by Hutten et al.; importantly this study highlighted the simultaneous expression of multiple inhibitory checkpoints (PD-1/TIGIT/KLRG-1) on antigen specific CD8 T-cells of the PB as a key feature of T-cell dysfunction [48].

#### CD47

*CD47* represents a macrophage immune checkpoint highly expressed on leukemic stem cells (LSCs) and AML cells, leading to immune evasion through the inhibition of phagocytosis [63]. In this process, CD47 functions as a “don’t eat me”-signal, preventing the recognition of the malignant T-cells by activated macrophages, neutrophils or dendritic cells (DCs) [64], whereat this mechanism could be involved in relapse of AML patients. Treatment with CD47-antibodies, such as Magrolimab (Hu5F9-G4) or Evorpacept (ALX148) showed promising effects on high risk MDS and AML in phase I/II clinical trials [65, 66], whereas a third antibody is currently under preclinical investigation [67].

#### CD200

Another surface molecule in this context is *CD200*, which is a new putative checkpoint on LSCs in AML and was significantly overexpressed on these cells [68]. Positivity for CD200 has been correlated with high relapse risk in AML [69]. A recent study reported the contribution of CD200 to immune escape in AML using BM and PB samples of humans and humanized mice models [70]. CD200 exerted an immunosuppressive function affecting the cytokine secretion and elimination capacity of T-cells. The CD200-mediated suppression was reversible when blocking the interaction with the CD200-receptor (CD200R) and a further study showed the benefit of a fully human CD200 antibody (TTI‐CD200), that improved immune responses to AML [71] indicating a therapeutic implication for relapsed AML patients.

#### Further receptors/ligands

*Further inhibitory immune checkpoint receptors/ligands* such as LAG-3, VISTA, 2B4, B7-H3, B7-H4, and LILRB4 [55, 72], and activating ones (ICOS, OX40, 41BB, CD70, CD28) [17] may be involved in the immune evasion process of AML after allogeneic transplantation, but their exact role remains still unclear.

In summary, the overexpression or upregulation of checkpoints on T-cells or the corresponding ligands on AML blasts has been reported in up to 40% of AML patients [17] who therefore might be the best candidates for therapy with ICB. An overview of checkpoints and ligands (potentially) involved in immune escape of AML and their role regarding relapse after allo-HCT is given in Table 1.

**Table 1:** Checkpoints (potentially) involved in AML relapse after allo-HCT

Checkpoint	Name	Expression	Impact on immune system regarding immune evasion	(Potential) Therapeutic intervention	Role in context of allo-HCT
PD-1 (CD279)	Programmed cell death protein 1	T-cells, B cells, NK-cells	Co-inhibitory signals, T cell exhaustion, T_reg_ recruitment	PD-1 antibodies (f.e. Nivolumab, Pembrolizumab) [73–75]	Association between high PD-1 expression and leukemia relapse [54, 55]
PD-L1 (CD274)	Programmed cell death 1 ligand 1	AML blasts	Co-inhibitory signals, T-cell exhaustion, T_reg_ recruitment	PD-L1 antibodies [76], silencing of PD-L1 by application of specific siRNA [77]	Highly upregulated in cases of relapse [17, 45]
TIM-3	T-cell immunoglobulin and mucin-domain containing-3	AML blasts, T-cells, T_regs_, NK-cells	Overexpression in AML on various cell types; increased levels in the plasma together with soluble TIM-3 lead to T-cell exhaustion and impaired NK-cell function	TIM-3 blockade [78], CAR-T-cells targeting TIM-3 [79]	Increased expression on CD8^+^ T-cells of relapsing patients [47, 54]
Gal-9	Galectin-9	T-cells, secreted by AML cells	Binds on TIM-3 and leads to IDO1 production; results in T-cell exhaustion and NK-cell dysfunction (see TIM-3)	Gal9 antibodies (shown to promote T-cell mediated killing of tumor cells, but not for AML so far) [80]	Unclear, may be involved in the TIM-3 immune escape mechanism
CTLA-4	cytotoxic T-lymphocyte-associated Protein 4	T-cells /T_regs_ AML blasts	Upregulation leads to T-cell inhibition and T-cell exhaustion	CLTA-4 antibody [81]	Increased levels in relapsing patients [47, 82]
CD80	CTLA-4 Counter-Receptor B7.1/ T-Lymphocyte Activation Antigen CD80	Antigen presenting cells (APCs), AML blasts	Provides costimulatory signal to T-cells (activation marker together with CD86)	-	upregulated in cases of relapse [17]; high expression correlates with low relapse-free survival [83]
TIGIT	T-cell Immunoreceptor With Ig And ITIM Domains	T-cells, T_regs_, NK-cells	Inhibits T-cell effector functions by binding CD155 or CD112, augments immunosuppressive function of T_regs_	TIGIT blockade [61]	higher expressed in the BM of relapsed AML patients after allo-HCT [55, 58, 72]
CD155 (PVR)	Poliovirus receptor, ligand for TIGIT	DCs, macrophages, T-cells, B cells, tumor cells (AML)	Inhibitory T-cell ligand; CD155-TIGIT pathway suppresses immune responses by secretion of IL-10 and reduced IL-12; induces a tolerogenic phenotype in T-cells, decreases NK-cell mediated tumor reactivity	PVR/PVRL2 antibodies [84]; Downregulation by FLT3-inhibition (FLT3 inhibitors) [85]	Potentially involved in immune escape of AML [62]
CD112 (PVRL2)	poliovirus receptor related 2, ligand for TIGIT	Tumor cells (AML)	CD112-TIGIT interaction leads to immune suppression	PVR/PVRL2 antibodies [84]; Downregulation by FLT3-inhibition (FLT3 inhibitors) [85]	upregulated in cases of relapse [17]
CD47	CD47 molecule (macrophage immune checkpoint)	LSC	Increased expression inhibits phagocytosis of leukemia cells	Humanized anti-CD47 [67, 86, 87]	highly expressed on LSCs, potentially involved in relapse of AML [63]
CD200	CD200 molecule	LSC	Overexpression results in immunosuppression of and impaired metabolic function of T-cells	CD200 antibody [71]	High risk of relapse for CD200^+^ AML patients [68–70]
KLRG-1	Killer Cell Lectin Like Receptor G1	Effector memory CD8 T-cells and NK-cells	Co-inhibitory checkpoint	KLRG-1 blockade (but not for AML so far) [88]	increased co-expression of KLRG-1 together with PD-1 and TIGIT during relapse after allo-HCT [72]
LAG-3	Lymphocyte-activation gene 3	T-cells, NK-cells, plasmacytoid DCs	co-inhibitory receptor, upregulated in AML	LAG-3 blockade (NCT04913922, AML patients)	Potential role in immune escape [89], but exact role unclear
OX40 (CD134)	Tumor necrosis factor receptor superfamily, member 4	T-cells, T_regs_	OX40 recepter interaction with its ligand OX40L leads to a costimulatory signal for T-cell proliferation	OX40 agonist monoclonal antibody [90]	OX40 positive T-cells were more frequent in AML than in healthy individuals [45]; high expression of OX40 associated with poor survival [91]; OX40 overexpression in relapsed AML, but potential role in immune escape unclear [92]
VISTA	V-domain Ig suppressor of T-cell activation	Neutrophils, monocytes, macrophages, DCs, T-cells, T_regs_, tumor cells	Induces an immunosuppressive environment, inhibitory towards T-cells	VISTA inhibitors (tested in clinical trials, but not for AML) [93]	VISTA expression levels at baseline correlated with disease recurrence; relapse after chemotherapy within 2 years from diagnosis had increased VISTA expression in leukemia and T-cells [55, 72, 94] role regarding relapse after allo-HCT still unclear
B7-H3 (CD276)	B7 homolog 3 protein	AML blasts from patients with monocytic AML	Attenuates cytotoxicity of NK-cells; stimulatory effect on Cd8 + cytolytic T-cell activity in AML; in contrast to this: B7-H3 reduces T-cell mediated Interferon release (exact role unclear)	B/-H3 targeted CAR T-cells; monoclonal antibodies [95]	upregulated in cases of relapse [17, 55, 72], high expression associated with poor prognosis
B7-H4	V-set domain-containing T-cell activation inhibitor 1	TAMs, antigen presenting cells (APCs)	T-cell coinhibitory molecule; negatively influences T-cell immune responses by binding on activated T-cells	Anti-B7-H4 [96] (not for AML so far)	Promotes immune escape potentially also in AML; exact role in AML relapse unclear [55, 72]
LILRB4	leukocyte immunoglobulin-like receptor-B 4	Highly expressed on monocytic AML cells (Leukemia blasts and LSCs); monocytes, macrophages, DCs and plasma cells	inhibitory checkpoint receptor, results in T-cell suppression via T_reg_ activation	Anti-LILRB4 CAR T-cell therapy [97]	Potentially involved in immune escape, but role is unclear so far

### Remodelling of the tumor microenvironment

The tumor microenvironment (TME) comprises a complex network of immune cells and non-cellular components. Malignant cells are able to change various physiological processes within the TME to their advantage. Hence, both immune effector cell dysfunction and alterations of the non-cellular components may contribute to tumor immune escape by remodelling of the TME towards an immunosuppressive phenotype.

#### T-cells dysfunction beyond HLA- and immune checkpoints

Beyond T-cell dysfunction associated with HLA loss/downregulation and ICP, other mechanisms leading to impaired T-cell reactivity in AML have been described.

The formation of an immunological synapse among T-cells and myeloid blasts is formed in a multistep process leading to adhesion between T-cell and antigen-presenting cell (APC) as well as T-cell and blasts. For this complex formation, the actin cytoskeleton and the polymerization of actin is critical [98]. Le Dieu et al. demonstrated impaired ability of T-cells from AML patients to arrange immune synapses with blasts, and restricted expression of genes important for the actin cytoskeleton [99]. Hence, AML blasts seem to alter the formation of immune synapses and thus prohibit correct communication between T-cells and blasts, although the responsible molecular processes have still to be investigated in detail.

Noviello et al. analysed the T-cell compartment of AML patients relapsing after allo-HCT and reported the presence of exhausted BM T-cells with restricted TCR repertoire and impaired T-cell effector functions, including reduced IL-2, γ-IFN and TNFα secretion and lower degranulation rates. Beyond, a small fraction of severely exhausted T_cms_,(characterized by PD-1, but in particular by the expression of the transcription factors T-bet and Eomesodermin, both regulators of T-cell exhaustion), was identified early after transplantation. This finding suggests limited and exhausted T-cell immunity early post HCT to be a strong risk factor for AML relapse and therefore part of the immune evasion process [47]. Remarkably, exhausted T-cells can be rescued, if they display an early, but not a late exhausted phenotype, with the therapeutic window being quite narrow [100].

Regulatory T-cells (T_regs_) control the immune homeostasis via induction of immunosuppression [101]. Numbers of T_regs_ are highly enriched in the blood and/or BM of myeloid leukemias after allo-HCT [102] and correlate with inferior outcome and post-transplant relapse [45, 103, 104]. Studies evaluating the immune landscape after-HCT showed similar frequencies of total T_regs_ in patients and healthy controls. However, T_regs_ from AML patients showed an activated phenotype (CD45RA^−^HLA-DR^+^) indicating a potential role of these cells in relapse [60]. Interestingly, results from another study indicate that host T_regs_ are not able to suppress the GvL effect in AML, and suggest that the predominance of these T_regs_ could even be a favourable prognostic marker [105]. These data identify T_regs_ as a kind of a double-edged sword, as they can have opposite effects promoting relapse and at the same time preventing the development of graft-versus-host disease (GvHD).

#### Impaired NK-cell activity

NK-cell mediated toxicity against malignant cells belongs to the major defence strategies of the immune system and is based on receptor-ligand interaction between NK-cell and tumor cell as well as lysis of tumor cells via antibody-dependant cell-mediated cytotoxicity (ADCC) [106, 107]. NK-cells exert anti-leukemic effects in the allo-HCT setting as they are the first lymphocytes to reconstitute after transplantation, and this successful recovery is related to reduced risk of relapse [108]. However, NK-cell escape by AML blasts can be caused by imbalanced receptor/ligand expression, which can occur in different ways. Epigenetic alterations in form of incorrect hypermethylation of the genes MICA, ULBP1, ULBP2, and ULBP3, which represent ligands for the activating NK-cell receptor NKG2D result in impaired function of these ligands and prevent NK-cell activation [109]. Further, the total absence of NKG2D ligands on AML leukemic stem cells [110], the release of a soluble NKG2D ligand resulting in downregulation of the corresponding receptor on NK-cells [111], reduced expression of activating receptors on NK-cells such as DNAM-1 (CD226) [112] and the induction of co-inhibitory receptors on NK-cells such as TIGIT [59], lead to impaired NK-cell activity. Even if these mechanisms have not been studied in the allo-HCT setting, it seems reasonable that they might be involved in immune escape of relapsing AML patients. Further investigation is strongly needed as NK-cell-based immunotherapy plays a major role in the context of HCT [113] including adoptive NK-cell transfer, CAR-NK-cell therapy (reviewed in [114]). Moreover, there is evidence that NK-cell function in relapsing AML undergoing allo-HCT can be restored [115] suggesting another potential therapeutic intervention for these patients.

#### Suppressive effects of leukemia-associated macrophages

Further cell types potentially contributing to immune escape are tumor-associated-macrophages (TAMs). TAMs are characterized by anti-inflammatory activity [116] and have been shown to be elevated in the BM of AML patients (referred to as leukemia-associated macrophages, LAMs) [117, 118]. As described in the previous section, it was shown that the checkpoints TIGIT, TIM-3 and LAG-3 were increased on LAMs of AML patients and this finding was associated with an intermediate or adverse genetic risk according to the European Leukemia Network (ELN) criteria [61]. AML blasts can produce Arginase II, which promotes the switch of macrophages from the M1 phenotype into a suppressive M2-like phenotype supporting immune evasion [119]. From the therapeutic point of view, apart from macrophage-targeted therapy via CD47 blockade (see section 2), more recent studies have reported the potential of chimeric antigen receptor macrophages (CARMs), which were able to create effective anti-tumor responses in haematological malignancies [120].

#### Alterations of the cytokine milieu

Hematologic tumors may alter the TME by switching cytokine production from pro-inflammatory to anti-inflammatory and by increasing the release of immunosuppressive molecules. As an example, the physiological function of IL-15 comprises the expansion and activation of effector T-cells and NK-cells and the promotion of memory T-cell generation. In the post-transplantation setting of different haematological malignancies low plasma levels of IL-15 have been associated with a higher risk of relapse [121]. In AML, this might be caused in part by an internal tandem duplication of the FLT3 tyrosine kinase in leukemic cells leading to reduced production of IL-15 mRNA. Remarkably, this phenomenon was reversible by application of the FLT3 tyrosine kinase inhibitor sorafenib, leading to an increase in CD8 + CD107 + IFNy + T-cells with antileukemic activity [122].

Apart from IL-15, further members of the cytokine network in the TME play a prominent role in the post-transplant setting, including the chemokine receptor CXCR4 and its ligand CXCL12. CXCR4 belongs to the group of transmembrane G-coupled protein receptors and is expressed on normal stem cells as well as AML blasts controlling the migration of LSCs to the BM [123]. Increased expression of this receptor on AML blasts has been correlated with increased risk of relapse and poor outcome, suggesting that the CXCR4/CXCL12 axis might be involved in immune escape of AML [124–127]. This is underlined by the ability of CXCR4/CXCL12 to activate pathways that target survival, growth and chemotherapy resistance of AML blasts [128].

Studies about chemokine profiles prior and post BM transplantation as recently performed for CXCL1, CXCL10 and CXCL12 [129], might identify further fundamental players of immune escape. Of special interest in this context is Interferon-γ (IFNγ), which is a key player of cellular immunity. In contrast to patients in remission, IFNγ secretion was strongly reduced in CD8^+^ T-cells from relapsing AML patients after allo-HCT, indicating an important role for this cytokine in immune evasion of AML [130]. Moreover, IFNγ can induce PD-L1 expression on AML blasts reflecting the dual nature of this cytokine as it can exert pro- and anti-tumoral immune responses [131]. A recent study demonstrated in the *ex-vivo* setting the impact of transforming growth factor beta 1 (TGF-β1), which induced NK-cell dysfunction in AML patients with early relapse after allo-HCT. This effect was reversible by pharmacologic inhibition of TGF-β1 signalling in leukemia xenograft mouse models [115], indicating a potential therapeutic role of TGF-β1 blockade as already shown in other cancer entities [132, 133].

#### Modulation of the TME by immunosuppressive enzymes

Closely linked to the cytokine milieu in the TME are metabolic active molecules that have been reported to modulate the TME in leukemia. One example is Arginase II, of which high plasma levels in AML patients, contributing to an immunosuppressive environment by blocking T-cell functions and inducing the suppressive M2-like phenotype of macrophages [119]. Hence, Arginase II might play a role in immune escape and relapse [8, 62]. Another potential player in this context is indoleamine 2,3-dioxygenase 1 (IDO1), which is expressed on AML blasts and is involved in the tryptophan degradation, finally leading to inhibition of T-cell proliferation, increased T-cell apoptosis and induction of T_regs_ [134]. IDO1 expression in leukemic cells has not been analysed in the post-transplant setting, but is convincingly associated with shortened relapse-free survival and overall survival in AML [135–138]. Two other enzymes currently discussed in the context of AML are CD39 and CD73 which belong to the family of ectonucleotidases involved in the degradation of adenosine triphosphate (ATP) [139]. In CD73-deficient mice after allo-HCT, low activity of CD73 improved the recognition and destruction of leukemic cells [140], indicating that CD73 is involved in tumor immune escape after transplantation.

Regarding other metabolic processes, glycolysis has been shown to play a critical role regarding immune evasion in AML: In patients relapsing after allo-HCT it was shown that in response to leukemia-derived lactic acid, the T-cells exhibited reduced glycolysis, functionally leading to decrease in proliferation and anti-leukemic activity. Sodium bicarbonate restored the metabolic fitness of donor T-cells both in vitro and in vivo after application to patients with AML relapse post-transplant receiving treatment with DLI [130].

#### Further immunosuppressive aspects of the tumor microenvironment

Related to cytokines and enzymes are the secreting cellular components, which increase the immunosuppressive phenotype of AML. Among these, T_regs_ and Myeloid-derived suppressor cells (MDSCs) play a role, which both exert an immunosuppressive function by damping anti-tumor T-cell responses [141]. A recent study analyzed the dynamics between MDSCs and T_regs_ of AML patients relapsing after allo-HCT [60] and showed that in contrast to constantly elevated T_reg_ levels, MDSCs subsets were increased early post-transplantation, but decreased over time. Nevertheless, no association between MDSCs and T_regs_ with subsequent relapse could be found [142].

Tumor infiltrating lymphocytes (TILs) comprise a heterogenous cluster of cells closely located to the tumor to create an anti-tumor response [143]. TILs have been shown to be associated with prognostic outcome and therapy response in several tumor entities and where found in 33–50% of the tested AML cohort [144, 145], although their presence alone was not sufficient for tumor elimination. However, their contribution to the prevention of immune escape remains unclear, as not a single report about TILs in relapsed AML patients after allo-transplantation exists.

Mesenchymal stroma cells (MSCs) have been shown to inhibit NK-cell-mediated killing of AML blasts and in general exhibit a more immunosuppressive phenotype than MSCs from healthy donors [146–148]. Post HCT MSCs showed differential mRNA expression, which normalized with disease remission [149], however, their role in immune escape of relapsed AML remains to be elucidated.

Finally, the vascular endothelium plays a major role for AML progression, as remodelling of the vascular compartment has been shown including high vascular permeability and reduced blood flow impairing the distribution of drugs and immune cells [150]. M2-macrophages are reported to be part of this process via promotion of blood vessel formation secreting pro-angiogenic cytokines such as the vascular endothelial growth factor (VEGFA) [151]. Vascular remodelling can be counteracted via genetic or chemical preservation of the endothelium, thereby improving clinical outcome of murine AML models [152].

**Fig. 1:**
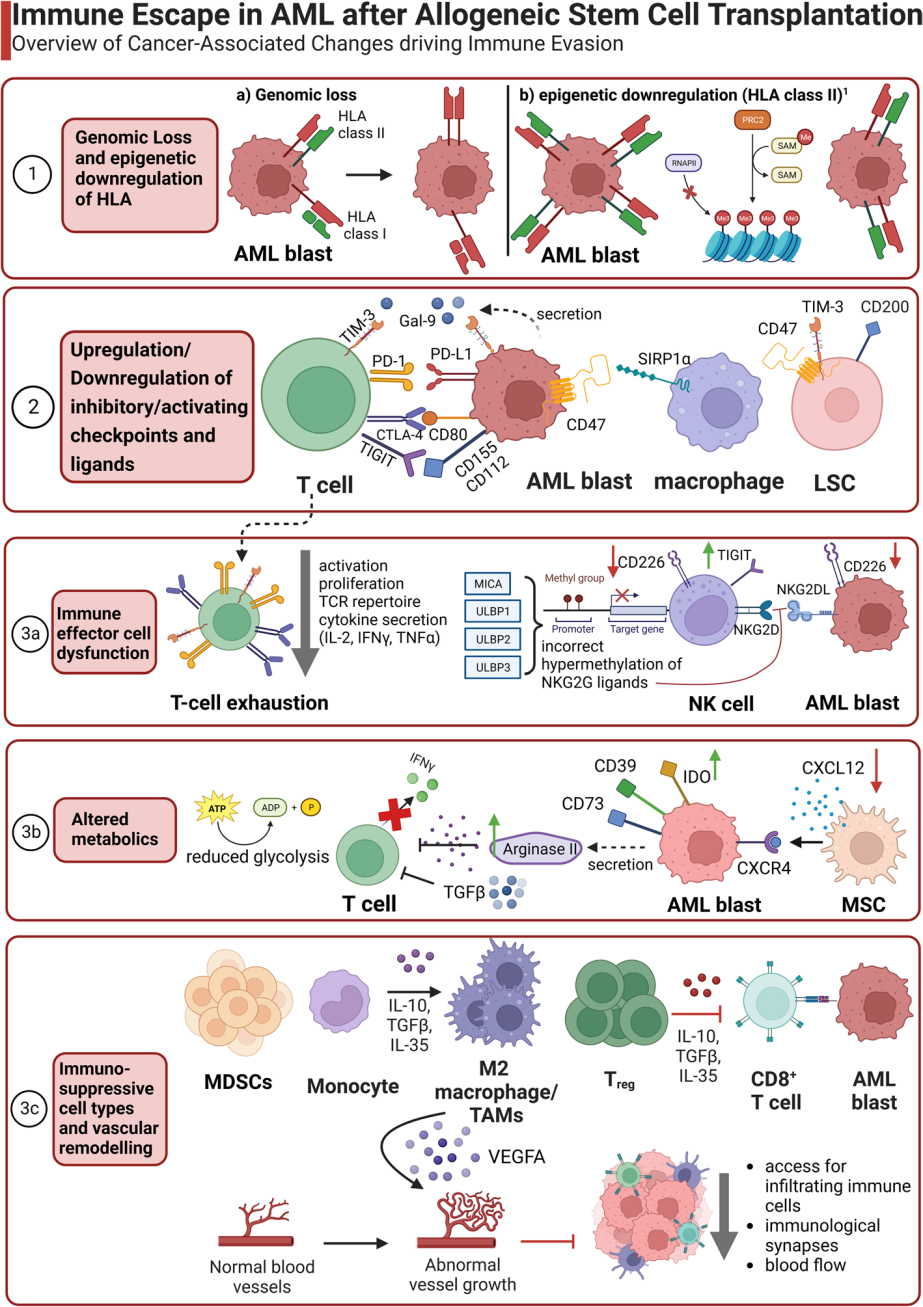
Overview of immune escape mechanisms in AML after allogeneic stem cell transplantation. The various immune evasion strategies include genomic loss or downregulation of HLA (1), upregulation of inhibitory or downregulation of activating receptors and the corresponding ligands (2), exhausted and dysfunctional immune effector cells (3a), an altered metabolic environment (3b) as well as an increase of immunosuppressive cell types (MDSCs, M2 macrophages and TAMs, T regs) and vascular remodelling of the AML niche cells (3c). This figure was created using Biorender. ^1^ Recent studies indicate that epigenetic downregulation could also affect HLA class I [26]. AML blast: acute myeloid leukemia blast, LSC: Leukemic stem cell, DC: dendritic cell, NK cell: Natural killer cell, MSC: mesenchymal stem cell, MDSC: myeloid derived suppressor cell, TAM: tumor associated macrophage. ↑ indicates upregulation, whereas ↓ marks downregulation

A summary of immune escape mechanisms described after allo-HCT for AML is depicted in Fig. 1.

### Therapeutic strategies targeting immune escape in relapsing AML

#### Current therapy

So far, no uniform standard treatment for AML relapse after allo-HCT has been defined, nor have approaches specifically addressing immune escape mechanisms become clinical routine. In principle, initial disease control is approached by either conventional chemotherapy, hypomethylation agents (HMA) ± venetoclax, or specific therapies targeting mutations (tyrosine kinases or IDH). However, subsequent cellular therapies relying on an allogeneic immune reaction – i.e. DLI or a second allogeneic HCT (allo-HCT2)—are regarded mandatory. [6, 153]. Allo-HCT2 has achieved long-term remission in about 30% of patients [154–158], and showed a trend for improvement over time [159]. However, its feasibility is strongly dependent on patient´s performance status and donor availability. DLI, either alone or in combination with other strategies, produced 2-year OS rates between 13–25% [6, 160, 161]. The role of DLI in high-risk malignancies has been recently reviewed [162]. Treatment options for patients not eligible for allo-HCT2 or DLI are limited and usually without curative potential [163]. Further details on the treatment of post-transplant relapse have recently been summarized [164].

Increasing knowledge on mechanisms of relapse have also allowed deeper insights into the interplay between the leukemia and the allogeneic immune system under currently used treatments. As an example, HMA have been shown to augment the GvL-effect by inducing cytotoxic CD8 + T-cell responses against tumor-associated antigens [165, 166], although upregulation of inhibitory immune checkpoints under HMA treatment has been associated with inferior outcome both in the transplant [167] and non-transplant setting [82]. With respect to DLI, reversion of T-cell exhaustion by downregulation of checkpoints has been proposed as a mechanism of the therapeutic efficacy [168–170]. Beyond, re-enforcement of the GvL effect in DLI recipients may be related to DLI-induced GvHD and the associated release of IFNγ, which has been shown to restore HLA-II expression on blasts [17, 171].

#### Experience from treatments addressing distinct immune escape mechanisms

So far, outside of clinical trials, the immune escape mechanisms described in this review are not yet routinely considered neither for treatment choice nor incorporation of specific elements into the management of post-transplant relapse. In this section we summarize published clinical experience from therapeutic approaches considering particular immune escape mechanisms.

##### Reversion of epigenetic HLA downregulation

When HLA class II downregulation was described as a mechanism of immune escape of AML after allo-HCT, it was evident, that this phenomenon was of epigenetic nature and therefore potentially reversible. As discussed in the previous paragraph, induction of IFNγ release e.g. as a consequence of DLI-induced GvHD might restore the expression of HLA II on blasts. In that sense, DLI addresses immune escape by reversion of HLA downregulation, a finding that is thought to explain in part the correlation of GvHD with GvL effects. Similarly, significant increase in the expression of MHC-II molecules and killing of leukemia cells was found to be mediated by IFNγ secretion from CD4 T-cells in a series of *in-vitro* experiments testing the activity of CD123-directed immunotherapies (flotetuzumab, a CD123xCD3 dual affinity retargeting protein [DART], as well as CAR T-cells directed against CD123) both in cell lines and xenograft models from patients with relapsed AML after allo-HCT with low baseline MHC-II expression [172]. Hence, beyond the direct antileukemic effects of the DART, respectively the CAR T cells, reversion of the HLA downregulation might contribute to the clinical effects of these compounds [173].

##### Therapeutic consequences drawn of HLA loss

In cases of HLA loss, the allogeneic effect mediated by T-cells is practically abrogated, as demonstrated by Vago et al. [14]. DLI or allo-HCT2 from a same donor is therefore expected to be ineffective, arguing for a change to a new donor with a different mismatched haplotype for allo-HCT2. This was supported by a small series of patients with AML relapse after haplo-HCT, in which a donor change for haplo-HCT2 improved clinical outcome [174], whereas this could not be shown in a recent retrospective analysis from the EBMT Acute Leukemia Working Party [158]. However, in both studies, HLA loss was not routinely tested. In contrast, Crucitti et al. [38] performed allo-HCT2 based on HLA loss analyses in a small group of patients, whereby donor change was associated with longer survival in patients with relapse involving HLA loss. Another finding supporting treatment guidance by HLA loss was reported by Muniz et al.: Three patients who had received DLI plus chemotherapy upon relapse post haplo-HCT were retrospectively identified to have HLA loss. All developed GvHD, but nevertheless did not show any response of their malignancy and died of disease progression [32]. Similarly, Wu et al. [34] also observed that in patients with minimal residual disease (MRD), the administration of pre-emptive DLI did not influence the median time to relapse in patients with HLA loss.

##### Treatment strategies addressing immune checkpoints

Immune checkpoint blockade (ICB) has shown activity in some hematologic disorders, especially lymphoid malignancies [43]. After allo-HCT, ICB is expected to reinvigorate the GvL effect; however simultaneously the activation of previously exhausted T-cells bears the risk of unleashing an uncontrollable alloreactivity causing life-threatening GvHD [175].

Davids et al. were among the first to explore the safety and efficacy of ICB after allo-HCT. In two prospective phase I trials they used ipilimumab [73] and nivolumab [176] to treat post-transplant relapse in various haematological malignancies including AML. In both trials, about 1/3 of patients responded. While patients with lymphoid malignancies seemed to benefit more, myeloid malignancies showed only modest response, except four patients with extramedullary disease, who achieved complete remission upon ipilimumab. Beyond, responses to ipilimumab were only observed after a higher dose (10 mg/kg). However, in both trials immune-related adverse events (IrAE) were considerable, including dose limiting toxicities and deaths due to GvHD of gut and liver as well as other IrAE (pneumonitis, colitis, and hepatitis). In a similar study, Godfrey et al. [74] evaluated the use of pembrolizumab in 12 patients with relapsed haematological malignancies, whereby only two achieved stable disease (SD). Various IrAE, but no GvHD, were observed. Finally, the combination of DLI and ICB has been used. As reported by Holderried et al., patients seemed to respond better than to nivolumab or ipilimumab alone, however at the cost of severe GvHD, which was observed in all patients receiving DLI plus nivolumab. Although response rate was higher with GvHD, this was counterbalanced by an increase in treatment-related mortality [177]. See Table 2 for details on efficacy and toxicities of studies evaluating ICB.

**Table 2:** Studies on treatment options addressing distinct immune escape mechanisms

References	Intervention	Rational			Study Population	Study Type	Best outcome	Toxicity
**Addressed mechanism: HLA downregulation**								
Toffalori et al. [17]	Systemic IFNy production via induction of GvHD	Upregulation of MHC II molecules via IFNy			AML cell lines	Preclinical model	n.a	n.a
Gambacorta et al. [18]	EZH2-inhibitors (e.g. Tazemetostat)	Pharmacological PRC2 inhibition with upregulation of HLA II molecule (independent of IFNy)			AML cell lines	Preclinical model	n.a	n.a
Christopher et al. [16]	IFN-y	Upregulation of MHC II molecules			AML cell lines	Preclinical model	n.a	n.a
Rimando et al. [178]	Flotetuzumab (CD123xCD3 DART)/ CD123 CAR-T-cells	Upregulation of MCH II molecules via INFy production by CD4 + T-cells			AML cell lines and xenografts from relapsed/ refractory AML patients	Preclinical model based on samples from patients with AML relapse post allo-HCT enrolled in a Phase I/II trial	n.a	n.a
Uy et al. [173]	Flotetuzumab (CD123xCD3 DART)	Upregulation of MCH II molecules via INF-y production			88 patients with r/r AML	Phase I/II trial	ORR 30% at the recommended Phase II dose (500 ng/kg)	Most common grade ≥ 3 AE were IRR/CRS (8%), cytopenias, hypophosphatemia, hypokalemia
Ho et al. [22]	MDM2-inhibition	MDM2-inhibition enhances T-cell mediated allogeneic immune response by upregulation of HLA-I and II molecules and increase of TRAIL-R1/2 on AML blasts			AML cell lines and xenografts	Preclinical model	n.a	n.a
References	Intervention				Study Population	Study Type	Best outcome	Toxicity
**Addressed mechanism: HLA loss**								
Crucitti et al. [38]	Donor change for allo-HCT2			23 patients with myeloid malignancies (AML, *n* = 20) relapse and HLA loss after allo-HCT		Retrospective study	Donor change associated with better survival (median OS 146 months vs 69 months) in HLA loss relapses, but not different from “classical relapses (152 vs 91 months)	Early transplant related mortality was high (41%)
Muniz et al. [32]	DLI/ second HCT			6 patients with HLA loss (myeloid malignancies, *n* = 2, lymphoid, *n* = 4)		Retrospective study	3 patients unsuccessfully treated with DLI (+ chemotherapy/ other drugs), 1 patient achieved CR for 18 months after allo-HCT2 from alternative donor but relapsed thereafter, 2 patients died of disease progression	All 3 Patients with DLI developed severe GvHD
Wang et al. [33]	DLI/ chemotherapy/ CAR-T			40 patients with HLA loss (6 without overt relapse). Myeloid malignancies, *n* = 27		Retrospective study	3 received DLI: PD, 2 IFNα: PD, 5 received BSC. 20 patients received DLI, targeted therapies, chemotherapy, CAR-T-cells with unsuccessful results. 3 achieved CR after chemotherapy + azacytidine, sorafenib CAR-T-cells, respectively) 2-y OS of patients with HLA loss not different from classical relapses (33% vs 29%)	NRM was lower in HLA loss patients than those with classical relapses (*p* = 0.035)
Wu et al. [34]	DLI/ chemotherapy/ CAR-T/ second HCT			54 patients with HLA loss (AML, *n* = 29, ALL, *n* = 25)		Retrospective study	Pre-emptive DLI in HLA loss (*n* = 14) vs classical relapses (*n* = 10) did not prevent relapse after MRD diagnosis Response rates after salvage therapy was similar in HLA loss and classical relapses (55% vs 45%) Allo-HCT2 was performed only in 2 patient2 with HLA loss	not reported
References	Intervention		Rational	Study Population		Study Type	Best outcome	Toxicity
**Addressed mechanism: immune checkpoints**								
Davids et al. [73]	CTLA-4 blockade (Ipilimumab)		Inhibition of CTLA-4 mediated T-cell inactivation	28 patients with relapsed hematological neoplasia after allo-HCT (AML, *n* = 12)		Phase I trial	ORR 32% (best responses at 10 mg/kg (7/22), no responses at 3 mg/kg, *n* = 6). 3/3 with leukemia cutis achieved CR	DLT at 3 mg/kg: GvHD gut/liver 1/6, IrAE 2/6, DLT at 10 mg/kg: GvHD 3/22, IrAE Grade ≥ 2 4/22 (colitis, pneumonitis, hepatitis)
Davids et al. [176]	PD-1 blockade (Nivolumab)		Reversal of T-cell exhaustion	28 patients with relapsed hematological neoplasia after allo-HCT (AML, *n* = 10, MDS = 7)		Phase I trial	ORR 32% (3/5 evaluable patients at 1 mg/kg, 5/20 at 0,5 mg/kg). No patient with eAML responder (*n* = 6)	DLT at 1 mg/kg 2/6 Sepsis, antiphospholipid syndrome) DLT at 0.5 mg/kg 4/22 (GvHD gut/liver, other liver toxicities)
Godfrey et al. [74]	PD-1 blockade (Pembrolizumab)		Reversal of T-cell exhaustion	12 patients with relapsed hematological neoplasia after allo-HCT (AML, *n* = 8)		Phase I/II trial	ORR 22% at fix dose pembrolizumab 200 mg (lymphoid malignancies only)	IrAE Grade 3–4 5/12 hemolysis, thrombopenia, hypothyroidism, pneumonitis), no GvHD
Holderried et al. [177]	PD-1/ CTLA-4 blockade with or without DLI		Reversal of T-cell exhaustion	21 patients with hematological malignancies other than Hodgkin lymphoma (AML/MDS, *n* = 12)		Retrospective study	ORR Nivolumab: 40%, Nivolumab plus DLI: 80%, Ipilimumab: 20%	Overall a/c GvHD: 48% (100% after Nivolumab plus DLI) Grade 3/4 aGvHD or moderate/extensive cGvHD: 29% (60% after Nivolumab plus DLI). IrAE were rare
Daver et al. [179]	PD-1 blockade (Nivolumab) + Azacytidine		Reversal of T-cell exhaustion/ inhibition of PDL-1 mediated resistance to Azacytidine	13 patients with relapsed AML after allo-HCT		Phase II trial	ORR 23% (Nivolumab 3 mg/kg)	Not reported separately for post-HCT
Garcia et al. [180]	CTLA-4 blockade + Decitabine		Inhibition of CTLA-4 mediated T-cell inactivation	25 patients with relapsed MDS/AML after allo-HCT		Phase I trial	ORR 20%, mostly at 10 mg/kg (3/6 with myeloid sarcoma achieved CR)	IrAE 44% (63% at Ipilimumab 10 mg/kg). No DLT at 3 and 5 mg/kg. 3 DLT at 10 mg/kg
Albring et al. [75]	PD-1 blockade (Nivolumab)		Reversal of T-cell exhaustion	3 patients with AML relapse after allo-HCT		Case series	2 patients with response (1 CR, 1 SD)	GvHD grades 3 and 2, respectively
Yao et al. [181]	PD-1 blockade (Tislelizumab) + Azacytidine		Reversal of T-cell exhaustion	1 patient with AML relapse after allo-HCT		Case report	Complete molecular remission	Lethal GvHD
Wong et al. [182]	PD-1 blockade (Nivolumab)		Reversal of T-cell exhaustion	1 patient with AML relapse after allo-HCT		Case report	Transient response (PR/SD)	No GvHD, IrAE suspected
Penter et al. [183]	PD-1 blockade (Nivolumab)		Reversal of T-cell exhaustion	1 patient with AML relapse after allo-HCT		Case report	Transient response (PR/SD)	Not reported
**Addressed mechanism: Lactic acid-induced metabolic and functional T-cell inhibition**								
Uhl et al. [130]	Sodium Bicarbonate (NaBi)		Reversal of LA-induced impairment of T-cell function	Murine and human AML cell lines; AML cells from 10 patients with relapsed AML after allo-HCT		Preclinical model	n.a	n.a

According to these findings, the risk/benefit ratio does not support the implementation of ICB for the general population with myeloid malignancies relapsing after allo-HCT. Hence, the identification of subgroups with a higher chance for response seems reasonable. Penter et al. [184] identified an increased T-cell infiltration within the TME, and significant differences in gene signatures involving T-cell activation and antigen receptor signalling in patients responding to ipilimumab vs. those who did not. Furthermore, they observed an upregulation of interferon-response genes and checkpoint molecules in patients responding only transiently, which may be associated with developing resistance to ICB. In the Nivolumab trial, a higher baseline PD-1 expression on circulating T_regs_ and CD4 cells was associated with response [176]. Penter et al. [183] analysed the cellular mechanism of response to PD-1 blockade using multidimensional single-cell technology in a patient with AML and augmented PD-L1 expression, which transiently responded to nivolumab. At baseline, CD8 T-cells showed features of exhaustion and senescence with high expressions of TIGIT, LAG-3, KLGR1, CD57 and downregulation of IL7RA and CD28. At response, an increase in the proportion of non-exhausted memory CD4^+^ T-cells and a decrease in exhausted CD8^+^ T-cells was observed.

Since upregulation of PD-L1 on leukemic cells might also be responsible for resistance to HMA treatment [82], ICB have been studied in combination with HMA to improve their efficacy. [179, 180]. Among patients relapsing after allo-HCT overall response was 20%. Immune profiling showed higher baseline frequencies of CD3, CD4-effector and CD8^+^ T-cells in BM of patients responding to azacytidine/nivolumab, and high CTLA-4 expression on CD4^+^ T-cells in non-responders, whereas the baseline expression of PD-L1 on blasts/ PD-1 on CD3^+^ T-cells was not predictive for response. Penter et al. performed integrative transcriptome-based analyses after treatment with decitabine/ipilimumab [185]. Among transplanted patients, an increased *T-cells to blast* ratio was a major determinant for responses, reinforcing the role of low disease burden for long-term remission to immunotherapy. In extramedullary AML, a high infiltration of regulatory T_reg_-cells and the CTLA-4 or PD-1 expression on tissue resident T-cells predicted the response to ipilimumab.

##### Cytokine-based approaches

Based on the role of IL-15 for the activation of immune effector cells and the observation of reduced plasma levels in patients relapsing after allo-HCT, recombinant IL-15 has been clinically studied in combination with NK-cell infusion. However, data were conflicting, since remission rates of 35% were observed in relapsed/refractory AML upon haploidentical NK-cell infusions plus IL-15 [186], whereas a more recent study reported that systemic IL-15 resulted in reduced clinical responses to allogeneic adoptive cellular treatment [187]. Although supported by pre-clinical evidence [122], no combined therapy comprising IL-15 and DLI have been reported so far. Similarly, the application of IFNy has not yet been studied systematically in humans.

A summary of preclinical and clinical studies on treatment options addressing distinct immune escape mechanism are listed in Table 2.

### New tools for relapse target identification: sequencing strategies and new modelling

Despite the increasing number of studies about relapsing AML after allo-HCT, further investigation is needed to achieve a deeper, more comprehensive understanding of the immune mechanisms behind post-transplant relapse, allowing for rapid analysis and individualized treatment. Rapidly evolving techniques, including multiomics factor analysis [16, 18, 188], whole-genome and whole-exome sequencing [189, 190] or targeted next-generation sequencing (tNGS) [191] have already identified new immune escape targets and specific gene signature in relapsed AML. Other sequencing platforms such as spatial transcriptomics and single cell sequencing revealed immune dysfunction signatures in AML patients, predicting unresponsiveness to checkpoint blockade treatment [192]. For remodelling the complexity and heterogeneity of relapsing AML in the post-transplant setting, especially for a realistic depiction of the TME including cytokines and surrounding cells, in vitro cell culture and murine *in-vivo* models became more and more popular. Advanced cell culture systems comprising 3D biomimetic scaffolds or organ-on-chip technologies may overcome the limitations of traditional cell culture models lacking TME cells or other external factors by mimicking the bone marrow environment. Hence, these assays provide optimized conditions for studying AML in the context of relapse and drug resistance [193, 194].

## Conclusion and outlook

AML relapse after allo-HCT remains a challenging problem. It is hoped that the rapidly increasing knowledge of immune escape mechanisms might allow to exactly define the particular mechanism(s) responsible for AML relapse in an individual patient and hence develop a specific, pathophysiology-based and individualized treatment strategy. This is of particular relevance since at least some immune escape mechanisms, such as downregulation of HLA and increase of immune checkpoints, seem to appear in a mutually exclusive way [28], which is why a one-size-fits-all strategy might not be possible.

For the time being, the clinical application of specific treatment options is still at the very beginning and has not made its way into clinical practice outside clinical trials. However, a broad variety of identified mechanisms seem to offer attractive opportunities for specific interventions. Among approaches to revert HLA downregulation, the application of IFNγ is investigated in a clinical trial (NCT04628338). Beyond, EZH2 inhibition appears particularly promising in this context. In contrast to the IFNγ-dependent strategies, re-establishing HLA expression via EZH2-inhibition was not associated with PDL-1 upregulation [18]. Comparable effects were achieved in-vitro by eight different EZH2-inhibitors, which are in part already available for compassionate use. However, clinical studies are still to be awaited, as it is the case for the use of the MDM2 inhibitors, which could be another effective strategy to increase (p53-dependant) HLA II expression. In the setting of HLA loss, change to an alternative donor for allo-HCT2 seems reasonable and has become clinical routine in many centers, although evidence from controlled studies is missing.

As discussed above, currently available ICB had only moderate effects against myeloid malignancies, which is why their use at least as monotherapy is unlikely to become a therapeutic option in the post-HCT setting. The combined role of several checkpoint ligands simultaneously expressed on blasts from the same patient [47], as well as the multi-functionality of their interactions might complicate their use as therapeutic targets. However, studies investigating factors associated with better response support the idea that ICB is in principle able to reinstate the T-cell mediated GvL effect by reverting T-cell exhaustion, and encourages baseline analyses of PD-1 status and other T-cell characteristics for potential selection of suitable candidates. The combination of ICB with HMA is a currently investigated option in several trials. Similar as for the PD-1/PD-L1 and CTLA-4 axis, TIM-3 can be targeted with specific monoclonal antibodies, and even CAR T-cells [78, 79, 195]. For the TIM3-ligand Gal-9, promising antibodies were developed and confirmed to promote T-cell cytotoxicity towards tumor cells, although not yet in the AML setting [80]. The pathway is also targeted in an ongoing phase III trial investigating the role of the TIM-3 directed antibody sabatolimab (MBG453) in MDS [196], and the combinations of a TIM-3 antibody with the MDM2-inhibitor HDM201 (NCT03940352) or HMA (NCT04623216) are also studied. Antibodies against the PVR/PVRL2/TIGIT axis seem another promising therapeutic option for AML [59, 62, 84, 85]. Last, a variety of studies is currently evaluating the role of the CD47 antibody Magrolimab in the post- transplant setting (e.g.: NCT05823480), based on the promising data seen in non-transplant patients [65, 66].

With respect to systemic application of cytokine-based therapies, clinical evidence is still extremely limited. Co-administration of IL-15 and immune effector cells clinical results have revealed conflicting data. Hence, although promising, refinement of current concepts is required for successful clinical use. An approach that is closer to clinical evaluation is the systemic application of sodium bicarbonate for metabolic reprogramming of allogeneic T-cells, which has been proven its feasibility in humans and may have the potential of reinforce the GvL effect by counteracting the AML-mediated metabolic and functional inhibition of allogeneic T-cells [130].

Given the broad variety of therapeutic toeholds, there is an enormous number of ongoing clinical trial in the field. To provide an overview on the range of current activities, ongoing clinical trials targeting immune escape mechanisms in the post-transplant setting are summarized in the online supplement (see Additional file 1, information drawn from https://clinicaltrials.gov/, upon August 8th, 2023.). Of note, about half of these trials investigate immune modulating principles as maintenance for prevention of relapse in high-risk patients, i.e. in a setting without highly proliferating leukemia, to allow more time for immune effects to develop. Beyond, also studies investigating different cellular therapy-based strategies are listed, whose detailed description is out of the scope of this review.

In conclusion, we are facing a fascinating era of increasing understanding of immune escape mechanisms that allow myeloid blasts to evade from the allogeneic control after allo-HCT. Whereas preclinical research will continue to elucidate further evasion mechanisms as well as how these different mechanisms may interact, ongoing and future clinical trials will have to identify those mechanisms that can be successfully addressed to provide rational and individualized treatment to patients with imminent or overt relapse after allo-HCT.

### Supplementary Information

**Additional file 1:** **Additional Table 1.** Ongoing clinical trials investigating pathomechanism-derived approaches post allo-HCT*.

## Data Availability

Not applicable.

## Abbreviations

ADCC: Antibody-dependant T-cell-mediated cytotoxicity
ALL: Acute lymphoblastic leukemia
Allo-HCT: Allogeneic hematopoietic stem cell transplantation
Allo-HCT2: Second allogeneic hematopoetic stem cell transplantation
AML: Acute myeloid leukemia
APC: Antigen-presenting cell
ATG: Antithymocyte globulin
B7-H3 (CD276): B7 homolog 3 protein
B7-H4: V-set domain-containing T-cell activation inhibitor 1
BCL-2: B-cell lymphoma 2
BM: Bone marrow
CAR: Chimeric antigen receptor
CARMs: Chimeric antigen receptor macrophages
CD200R: CD200-receptor
CD80: T-Lymphocyte Activation Antigen CD80
CIITA: Class II Major Histocompatibility Complex Transactivator
CIML: Cytokine-induced memory-like
CR: Complete response
CTLA-4: Cytotoxic T-lymphocyte-associated protein 4
DART: Dual affinity retargeting protein
DC: Dendritic cell
DLI: Donor lymphocyte infusion
eAML: Extramedullary AML
EZH2: Enhancer Of Zeste 2 Polycomb Repressive Complex 2 Subunit
FOXP3: Forkhead box protein P3
Gal-9: Galectin-9
GM-CSF: Granulocyte–macrophage colony-stimulating factor
GvHD: Graft-versus-host disease
GvL: Graft-versus-leukemia
Haplo-HCT: Haploidentical allo-HCT
HLA: Human leukocyte antigen
HMA: Hypomethylating agens
ICB: Immune checkpoint blockade
IDO1: Indoleamine 2,3-dioxygenase 1
IFNγ: Interferon-gamma
irAE: Immune-related adverse event
JAK-2: Janus kinase 2
KLRG-1: Killer Cell Lectin Like Receptor G1
LAG-3: Lymphocyte-activation gene 3
LAMs: Leukemia associated macrophages
LILRB4: Leukocyte immunoglobulin-like receptor-B 4
LSC: Leukemic stem cell
MDS: Myeloid dysplastic syndrome
MDSCs: Myeloid-derived suppressor cells
miHag: Minor histocompatibility antigens
MMUD: Mismatched unrelated donor
mRNA: Messenger RNA
MSCs: Mesenchymal stroma cells
MSD: Matched sibling donors
MUD: Matched unrelated donor
Ng: Nanogram
ORR: Objective response rate
OS: Overall survival
OX40 (CD134): Tumor necrosis factor receptor superfamily, member 4
PB: Peripheral blood
PCR: Polymerase chain reaction
PD-1: Programmed cell death protein 1
PD-L1: Programmed cell death 1 ligand 1
PR: Partial response
PRC2: Polycomb repressive complex 2
Pt-CY: Post-transplant cyclophosphamide
PVR: Poliovirus receptor
r/r: Relapsed/ refractory
SCF: Stem cell factor
SD: Stable disease
TAA: Tumor-associated antigens
TAMs: Tumor-associated-macrophages
T_cms_: Memory stem cell
TCR: T-cell receptor
TIGIT: T-cell immunoreceptor with Ig and ITIM domains
TILs: Tumor infiltrating lymphocytes
TIM-3: T-cell immunoglobulin and mucin domain 3
TKIs: Tyrosine kinase inhibitor
TME: Tumor microenvironment
TNFα: Tumor necrosis factor alpha
TRAIL: Tumor Necrosis Factor Related Apoptosis Inducing Ligand
T_regs_: Regulatory T-cells
UCB: Unrelated cord blood units
VISTA: V-domain Ig suppressor of T-cell activation
