# Supplementary material for: Relapse of acute myeloid leukemia after allogeneic stem cell transplantation: immune escape mechanisms and current implications for therapy
Source: Mol Cancer. 2023 Nov 11;22:180. doi: 10.1186/s12943-023-01889-6 (PMC10640763; doi:10.1186/s12943-023-01889-6)
Supplement: Supplementary file 1 — Additional file 1: Additional Table 1. Ongoing clinical trials investigating pathomechanism-derived approaches post allo-HCT*. [file 12943_2023_1889_MOESM1_ESM.docx]

**Additional Table 1:** Ongoing clinical trials investigating pathomechanism-derived approaches post allo-HCT *

| **Addressed mechanism: HLA downregulation** | | | | | | | | |
| --- | --- | --- | --- | --- | --- | --- | --- | --- |
| Trial name | ClinicalTrials.gov ID | Drug/ Intervention | Population | Phase | Study Sites | Status | Study start | Estimated primary completion |
| Post-transplant Flotetuzumab for AML | NCT05506956 | Flotetuzumab | AML relapse after allo-HCT | Phase I | John Hopkins University | Recruiting | 2022.10.20 | 2025.12 |
| Flotetuzumab for Relapsed Acute Myeloid Leukemia (AML) and Myelodysplastic Syndrome (MDS) Following Allogeneic Hematopoietic Cell Transplantation | NCT04582864 | Flotetuzumab, DLI | AML relapse after allo-HCT | Phase II | Washington University | Recruiting | 2021.05.20 | 2026.01.31 |
| A Study of Siremadlin Alone and in Combination With Donor Lymphocyte Infusion in Acute Myeloid Leukemia Post-allogeneic Stem Cell Transplant | NCT05447663 | Siremadlin, DLI | AML in CR after allo-HCT | Phase Ib/II | United States, Australia, Germany, Israel, Italy, Spain, United Kingdom | Recruiting | 2023.02.23 | 2027.07.20 |
| IFN-γ to Treat Acute Myeloid Leukemia (AML) and Myelodysplastic Syndrome (MDS) That Has Relapsed After Allogeneic Hematopoietic Stem Cell Transplantation | NCT04628338 | IFNγ (interferon gamma-1b) injection with subsequent DLI | AML/MDS relapse after allo-HCT | Phase I | University of Pittsburgh | Recruiting | 2021-03-08 | 2023-06 |
| Palbociclib and Tazemetostat in Combination With CPX-351 for the Treatment of Relapsed or Refractory Acute Myeloid Leukemia | NCT05627232 | Palbociclib, Tazemetostat, CPX-351 | R/R AML (Age ≥ 18 year) including relapse post allo-HCT | Phase I | Jefferson University Hospital, Philadelphia, Pennsylvania | Not yet recruiting | 2023-08 | 2026-01 |
| **Addressed mechanism: immune checkpoints** | | | | | | | | |
| Trial name | ClinicalTrials.gov ID | Drug/ Intervention | Population | Phase | Study Sites | Status | Study start | Estimated primary completion |
| PD-1 Inhibitor, Azacytidine and Low-dose DLI in AML Relapse After Allo-HSCT | NCT05772273 | Camrelizumab, Azacytidine, DLI | AML relapse after allo-HCT | Not applicable | The First Affiliated Hospital of Soochow University | Recruiting | 2023.03.15 | 2024.07.01 |
| Sabatolimab as a Treatment for Patients With Acute Myeloid Leukemia and Presence of Measurable Residual Disease After Allogeneic Stem Cell Transplantation. | NCT04623216 | Sabatolimab (anti-TIM-3 antibody), Azacytidine | AML in CR after allo-HCT with MRD | Phase Ib/II | France, Germany, Italy, Spain | Recruiting | 2021.09.14 | 2024.10.09 |
| Magrolimab in Combination With Azacytidine After Allogeneic HCT in Treating Patients With High-Risk AML or MDS | NCT05823480 | Magrolimab (Anti-CD47 Antibody), Azacytidine | AML/MDS, Maintenance Therapy after allo-HCT | Phase I | City of Hope Medical Center (Duarte, California) | Recruiting | 2023.09.09 | 2025.05.03 |
| Nivolumab and Ipilimumab After Donor Stem Cell Transplant in Treating Patients With High Risk Refractory or Relapsed Acute Myeloid Leukemia or Myelodysplastic Syndrome | NCT03600155 | Nivolumab, Ipilimumab | AML/MDS relapse after allo-HCT | Phase I | MD Anderson Cancer Center | Recruiting | 2018.10.11 | 2024.03.31 |
| Relatlimab With Nivolumab and 5-Azacytidine for the Treatment of AML (AARON) | NCT04913922 | Azacytidine, nivolumab (anti-PD1 antibody), relatlimab (anti-LAG3 antibody) | R/R AML (18 years and older, including relapse 100 days post allo-HCT), newly diagnosed AML (Age >65 years) | Phase II | Ludwig-Maximilians - University of Munich | Recruiting | 2021.05.05 | 2025.03 |
| Pilot Study of Pembrolizumab Treatment for Disease Relapse After Allogeneic Stem Cell Transplantation | NCT02981914 | Pembrolizumab (anti PDL-1 antibody) | AML, MDS or mature B cell lymphomas relapsed after allo-HCT | Early Phase I | University of Chicago | Recruiting | 2017.03.07 | 2023.02 |
| Study of KITE-222 in Participants With Relapsed/​Refractory Acute Myeloid Leukemia | NCT04789408 | ITE-222, (autologous Anti-CLL-1 CAR T-cell Therapy) | R/R Acute Myeloid Leukemia including relapse post allo-HCT | Phase I | United States, France | Recruiting | 2021-07-19 | 2024-01 |
| Dose-Escalation and Dose-Expansion Study to Evaluate the Safety and Tolerability of Anti-CD7 Allogeneic CAR T-Cells (WU-CART-007) in Patients With CD7+ Hematologic Malignancies | NCT05377827 | Anti-CD7 Allogeneic CAR T-Cells (WU-CART-007) | Patients with CD7+ hematologic malignancies (including AML relapse post allo-HCT) | Phase I | Washington University School of Medicine | Not yet recruiting | 2023-07-31 | 2026-01-31 |
| **Addressed mechanism: Immune effector cells** | | | | | | | | |
| Trial name | ClinicalTrials.gov ID | Drug/ Intervention | Population | Phase | Study Sites | Status | Study start | Estimated primary completion |
| Gamma Delta T-cell Infusion for AML at High Risk of Relapse After Allo HCT | NCT05015426 | Artificial Antigen Presenting cell (AAPC)-expanded donor γδ T--Cell Infusion | AML in CR at High Risk of Relapse After Allo HCT | Phase I | H. Lee Moffitt Cancer Center and Research Institute | Recruiting | 2022.03.21 | 2024.12.31 |
| Efficacy of MT-401 in Patients With AML Following Stem Cell Transplant (ARTEMIS) | NCT04511130 | Donor-Derived Multi-Tumor-Associated Antigen Specific T-cells (MT-401, zedenoleucel) manufactured under Good Manufacturing Practice (GMP) | AML in CR post allo-HCT or relapse after allo-HCT (including MRD) | Phase II | University of Alabama | Recruiting | 2020.10.14 | 2024.07 |
| Administration of Donor Multi TAA-Specific T-cells for AML or MDS (ADSPAM) | NCT02494167 | Derived Multi-Tumor-Associated Antigen (TAA)- Specific T-cells | AML/MDS after allo-HCT (in CR or relapse) | Phase I | Baylor College of Medicine, Houston, Texas | Recruiting | 2016-02 | 2024-04 |
| Multi-institutional Prospective Research of Expanded Multi-antigen Specifically Oriented Lymphocytes for the Treatment of VEry High Risk Hematopoietic Malignancies (RESOLVE) | NCT02203903 | Tumor associated antigen lymphocytes (TAA-T) | AML/MDS after allo-HCT (in CR or relapse) | Phase I | John Hopkins University | Recruiting | 2015-01-01 | 2024-11-30 |
| Efficacy and Effectiveness of Adoptive Cellular therapy with Ex-Vivo Expanded Allogeneic γδ T-lymphocytes (TCB-008) for Patients With Refractory or Relapsed Acute Myeloid Leukaemia (AML) (ACHIEVE) | NCT05358808 | Ex-Vivo Expanded Allogeneic γδ T-lymphocytes (TCB-008) | AML relapse after allo-HCT | Phase II | United Kingdom | Recruiting | 2022-08-15 | 2023-05 |
| IS-free Treg HaploHCT | NCT04678401 | Immunosuppression-free regulatory T-cell Graft-engineered Haploidentical Hematopoietic Cell Transplantation | Relapsed/Refractory and Ultra-High-risk AML/MDS | Phase I | Dana-Farber Cancer Institute | Recruiting | 2021-01-12 | 2025-10-31 |
| Dendritic Cell/​AML Fusion Cell Vaccine Following Allogeneic Transplantation in AML Patients | NCT03679650 | Dendritic Cell/AML Fusion Cell Vaccine Alone and in Conjunction With Decitabine | AML, day 45-75 post allo-HCT | Phase I | Beth Israel Deaconess Medical Center | Recruiting | 2018.10.11 | 2023.08.31 |
| PLAT-08: A Study Of SC-DARIC33 CAR T-cells In Pediatric And Young Adults With Relapsed Or Refractory CD33+ AML | NCT05105152 | SC-DARIC33 (autologous T cell product that has been genetically modified to express a Dimerizing Agent Regulated Immunoreceptor Complex (DARIC) | R/R CD33+ AML (including relapse post allo-HCT, Age up to 30 years) | Phase I | Seattle Children´s Hospital | Recruiting | 2021-11-29 | 2021-11-29 |
| Study of Anti-CD33 Chimeric Antigen Receptor-Expressing T-cells (CD33CART) in Children and Young Adults With Relapsed/​Refractory Acute Myeloid Leukemia | NCT03971799 | CD33CART (Anti-CD33 Chimeric Antigen Receptor-Expressing T-cells) | R/R CD33+ AML (including relapse post allo-HCT, Age up to 35 years) | Phase I/II | Center for International Blood and Marrow Transplant Research | Recruiting | 2020-01-08 | 2024-12 |
| Safety and Efficacy of Allogenic NK-cells in Combination With Chemotherapy in the Treatment of r/​r AML After Allo-HSCT | NCT05744440 | Allogeneic NK-cells (JD002) | AML relapse after allo-HCT | Early Phase I | Xuzhou Medical University | Recruiting | 2023.03.01 | 2025.05 |
| Expanded Natural Killer Cells Following Haploidentical HSCT for AML/​MDS | NCT03300492 | *In-vitro* Expanded Natural Killer Cells | AML after Haplo-HCT (days +10,+15 and +20) | Phase I/II | University Hospital, Basel, Switzerland | Recruiting | 2018-11-12 | 2023-01-31 |
| Cytokine Induced Memory-like NK Cell Adoptive Therapy for Relapsed AML After Allogeneic Hematopoietic Cell Transplant | NCT03068819 | Cytokine Induced Memory-like NK Cell Infusion | AML relapse after allo-HCT in children and adults | Phase I/II | Washington University School of Medicine | Recruiting | 2017-10-23 | 2026-10-20 |
| Natural Killer Cell Immunotherapy in Combination With PARP-inhibition to Overcome NKG2D Mediated Immune Evasion in Acute Myeloid Leukemia | NCT05319249 | NK-cells, Talazoparib 1 MG [Talzenna] | AML after at least one line of AML therapy including relapse post allo-HCT | Phase I/II | University of Heidelberg | Not yet recruiting | 2023-06 | 2027-06 |
| A Trial to Evaluate the Safety and Efficacy of oNKord® in Subjects With Acute Myeloid Leukemia | NCT04632316 | oNKord® (an off-the-shelf, ex vivo-cultured allogeneic NK cell preparation) | AML in CR with MRD who are currently not proceeding to allo-HCT | Phase I/IIa | Belgium, France, Germany, Netherlands, Switzerland | Recruiting | 2020-12-08 | 2023-04 |
| **Addressed mechanism: Remodelling of the TME** | | | | | | | | |
| Trial name | ClinicalTrials.gov ID | Drug/ Intervention | Population | Phase | Study Sites | Status | Study start | Estimated primary completion |
| Relapse Prophylaxis With N-803 for AML and MDS Pts Following Allo HSCT | NCT02989844 | IL-15 super-agonist complex (N-803 formerly known as Alt-803) | AML/MDS following RIC allo-HCT | Phase II | University of Minnesota | Completed | 2017.04.12 | 2022.03.19 |

***** source: <https://clinicaltrials.gov/>, latest update 08.08.2023.
